# Ractopamine under dietary protein adjustment improves growth performance and carcass traits of immunocastrated and surgically castrated pigs slaughtered 21 days after vaccination

**DOI:** 10.1007/s11250-026-05271-8

**Published:** 2026-08-04

**Authors:** Diego Duran Araujo, Matheus de Almeida Ferreira, Lucas Alves Rodrigues, Idael Matheus Góes Lopes, Marcelo Dourado de Lima, Luíza de Almeida Ramos, Rafaela Jorge Sarsur de Freitas Ribeiro, Hemille Antunes Ferreira Miranda, Isabel Luísa Ribeiro de Abreu Teixeira, Francisco Carlos de Oliveira Silva, Walter Motta Ferreira, Dalton de Oliveira Fontes

**Affiliations:** 1 Department of Animal Science, Federal University of Minas Gerais (UFMG), Belo Horizonte, Minas Gerais Brazil; 2 Prairie Swine Centre, Inc., Saskatoon, Canada; 3 Agricultural Research Institute of Minas Gerais, Viçosa, Minas Gerais Brazil

**Keywords:** Feed intake, GnRH, Swine, β-adrenergic agonist

## Abstract

The objective of this study was to evaluate the effects of immunocastration and ractopamine hydrochloride (RAC) supplementation on growth performance and carcass traits of male pigs slaughtered 21 days after the second immunization. Sixty-four pigs (Landrace × Large White, initial body weight 103.05 ± 0.67 kg) were individually tagged and randomly assigned to a 2 × 2 factorial arrangement in a completely randomized design, consisting of two castration methods (immunocastrated vs. surgically castrated) and two dietary RAC levels (0 or 10 ppm). No castration method × RAC interactions were observed for any variable (*P* > 0.05). Immunocastrated (IM) pigs exhibited 19.5% greater average daily gain (ADG) and 13% lower feed-to-gain ratio (FG) compared with surgically castrated (SC) pigs (*P* < 0.05). IM pigs also showed reduced backfat thickness across all measurement sites and greater lean meat percentage, whereas SC pigs had higher carcass yield (*P* < 0.05). Ractopamine supplementation under increased amino acid supply increased ADG by 13.6%, improved hot carcass weight by 3.1%, and reduced FG by 13% (*P* < 0.05). Overall, immunocastration improved growth performance and carcass leanness, while RAC supplementation increased average daily gain by 13.6%, confirming its additive effect under a shortened 21-day post-immunization interval. Immunization against GnRH combined with RAC feeding represents an effective strategy for producing heavy-weight finishing pigs with improved efficiency.

## Introduction

Given the steady expansion of the global population and the parallel rise in demand for animal-derived protein, swine production has been required to increase biological efficiency without disregarding welfare standards or product quality. In this context, technological innovations and precision approaches have become increasingly important tools for improving productivity, efficiency, and sustainability in animal production systems (Mustafa et al. 2026). Current production models must reconcile productivity, carcass value, and economic viability within increasingly restrictive regulatory frameworks.

Among the strategies adopted in recent years, anti-GnRH immunization has emerged as a practical alternative to surgical castration, combining productive advantages with welfare considerations (Morales et al. 2017). By temporarily suppressing testicular function, immunocastration allows animals to express growth patterns similar to entire males while minimizing boar taint and avoiding the invasive nature of surgical procedures (Batorek et al. 2012; Han et al. 2019; Rossi et al. 2022). As a result, its use has progressively increased in commercial systems seeking technical and ethical alignment.

Ractopamine hydrochloride (RAC), in turn, remains widely incorporated into finishing diets in Brazil, particularly in intensive systems. Through β-adrenergic stimulation, ractopamine redirects nutrients toward lean tissue deposition, enhancing protein accretion and limiting lipid synthesis (Abbas et al. 2022). These effects on nutrient partitioning and carcass composition have been recognized for decades. Adeola et al. (1990) demonstrated that ractopamine supplementation increased lean tissue deposition and improved carcass composition in finishing pigs, establishing the biological basis for its use as a growth-promoting technology. Improvements in average daily gain, feed efficiency, and carcass leanness have been consistently reported with its use (Almeida et al. 2010; Abreu et al. 2024; Sánchez-Villalba et al. 2023). However, responses to RAC may vary according to physiological stage and endocrine status, which become especially relevant in immunocastrated males.

Although both technologies are consolidated in commercial practice, their combined effects during the short interval immediately following the second immunization dose remain insufficiently explored. Most available studies have evaluated pigs slaughtered 28 days or more after the second vaccination, whereas information regarding productive responses within a 21-day interval remains comparatively limited. Lealiifano et al. (2011) demonstrated that reducing the interval between the second anti-GnRH immunization and slaughter improved growth performance while maintaining effective control of boar taint compounds, highlighting the importance of evaluating productive responses during this specific post-immunization period. In contrast, commercial logistics and regulatory conditions frequently permit slaughter at 21 days.

Evidence indicates that immunocastrated pigs slaughtered after longer intervals show progressive increases in fat deposition and backfat thickness, approaching the carcass profile of surgically castrated males as testicular steroid production is suppressed (Elsbernd et al. 2017; Morales et al. 2017; Schiavon et al. 2022). The 21-day interval corresponds to a period of intense metabolic and behavioral adjustments, including increased feed intake and endocrine modulation (Elsbernd et al. 2017), factors that may influence responsiveness to anabolic agents such as ractopamine.

From a production standpoint, the interval between the second immunization and slaughter may influence the productive and carcass responses of immunocastrated pigs, particularly during the period of metabolic and endocrine adjustments that follows the second vaccination dose (Elsbernd et al. 2017; Morales et al. 2017). Understanding the biological responses occurring within this management window is important for optimizing the use of immunocastration under commercial production conditions. Additionally, carcass composition and meat quality in heavy pigs are influenced by animal category, metabolic condition, and pre-slaughter management, with stress sensitivity contributing to variability in final product traits (Sardi et al. 2020a, b). Under current commercial conditions, evaluating performance and carcass traits within this reduced interval is therefore technically relevant (Schiavon et al. 2022).

In this context, the present study examines the interaction between immunocastration and ractopamine supplementation in pigs slaughtered 21 days after the second vaccine dose, reflecting practical field conditions. Data addressing this specific management window remain limited, particularly when both strategies are applied simultaneously. Thus, the objective of this study was to evaluate growth performance and carcass characteristics of immunocastrated and surgically castrated males slaughtered 21 days after the second immunization when fed diets with or without ractopamine supplementation under nutritionally adjusted feeding conditions.

## Materials and methods

### Ethics declaration

All procedures were approved by the Animal Use Ethics Committee (CEUA) of the of the Agricultural Research Company of Minas Gerais (EPAMIG) under protocol number 03/2020.

### Experimental site, animals, experimental design, and treatments

The study took place at the Dr. Henrique Guimarães Fernandes Experimental Center (Granja São Francisco), Martinho Campos, Minas Gerais, Brazil. The facilities consisted of masonry barns with natural ventilation and no environmental control, representing commercial production conditions.

A total of 64 male pigs (Landrace × Large White), with an initial body weight of 103.05 ± 0.67 kg, were evaluated; 32 were immunocastrated and 32 surgically castrated. After individual identification, animals were assigned to a completely randomized design in a 2 × 2 factorial scheme, including two castration methods (immunocastration or surgical castration) and two dietary ractopamine levels (0 or 10 ppm/kg of feed). Each treatment comprised eight replicates, with two pigs per pen (Table 1).

**Table 1:** Distribution of animals according to treatment

Treatments	Sexual category	Ractopamine level (ppm/kg feed)
T1	Immunocastrated	10
T2	Immunocastrated	0
T3	Surgically castrated	10
T4	Surgically castrated	0

### Facilities and management

The animals were housed in eight pens distributed in four rows within a masonry barn. Each pen measured 4.84 m², had a partially slatted floor, one swing feeder (20 × 110 cm), and a nipple drinker. Feed and water were offered ad libitum throughout the trial. Ambient temperature was monitored daily using a thermometer positioned at shoulder height in the center of the barn to characterize the environmental conditions during the experimental period. Because all animals were housed simultaneously in the same facility and exposed to the same environmental conditions, temperature was not considered a treatment-related source of variation and was therefore not included in the statistical model.

### Castration procedures and immunization protocol

Surgical castration was performed at 7 days of age. Animals in the immunocastrated group remained intact until vaccination. Immunization used a commercial anti-GnRH vaccine (Vivax^®^) administered in two doses: the first at 106 days and the second at 140 days of age. Each 2-mL dose contained 0.4 mg of GnRF–protein conjugate. Injections were applied subcutaneously behind the ear in accordance with the manufacturer’s guidelines.

### Diets and feeding management

The experimental diets were formulated based on corn and soybean meal to meet or exceed the nutritional requirements of finishing castrated male pigs, according to the recommendations of Rostagno et al. (2017). Diets were formulated to be isonutritive, differing only by the inclusion of ractopamine hydrochloride (0 or 10 ppm). Levels of metabolizable energy, crude protein, digestible amino acids, minerals, and vitamins were kept constant across treatments. Synthetic amino acids, vitamins, and minerals were added as needed to ensure adequate nutrient balance, and diet composition and calculated nutritional values are presented in Table 2. The ingredient composition and nutritional profile of the diets are presented in Table 2. Throughout the experimental period, feed and water were provided ad libitum.

**Table 2:** Calculated composition of experimental diets

Ingredients	Diet without RAC	Diet with RAC
Ground corn	76.97	71.71
Soybean meal (46%)	18.20	22.48
Semi–whole soy (41%)	1.00	1.97
Dicalcium phosphate	1.00	0.97
Limestone	0.83	0.73
L-Lysine	-	0.02
L-Methionine	-	0.038
L-Threonine	-	0.025
L-Tryptophan	-	0.005
Vitamin–mineral premix^1^	2.00	2.00
Ractopamine (2%)^2^	-	0.05
Calculated Nutritional Levels		
Crude protein (%)	15	17
Metabolizable energy (Kcal/kg)	3.350	3.350
Digestible lysine (%)	0.700	0.850
Digestible methionine (%)	0.213	0.255
Digestible Met + Cys (%)	0.445	0.511
Digestible threonine (%)	0.462	0.552
Digestible tryptophan (%)	0.140	0.170
Calcium (%)	0.785	0.750
Available phosphorus (%)	0.380	0.380

¹ Provided per kg of diet: vitamin A (min.) 5005 IU, vitamin B1 (min.) 0.734 mg, vitamin B12 (min.) 0.015 mg, vitamin B2 (min.) 2.736 mg, vitamin B6 (min.) 1.468 mg, vitamin D3 (min.) 1101 IU, vitamin E (min.) 30 IU, vitamin K3 (min.) 2.169 mg, biotin (min.) 0.073 mg, choline (min.) 146.8 mg, nicotinic acid (min.) 21.7 mg, pantothenic acid (min.) 11 mg, copper (min.) 8.42 mg, iron (min.) 46.7 mg, iodine (min.) 0.577 mg, manganese (min.) 23.4 mg, selenium (min.) 0.210 mg, sodium (min.) 1900 mg, zinc (min.) 63.4 mg. ² Ractopamine was added to a concentration of 10 ppm/kg feed

Diets containing ractopamine were formulated with greater amino acid density to support the increased protein accretion associated with β-adrenergic stimulation. It is well established that ractopamine enhances lean tissue deposition and increases the requirement for digestible amino acids, particularly lysine, due to its effects on muscle protein synthesis and nutrient partitioning (Apple et al. 2004). Therefore, the nutritional adjustment adopted in the present study aimed to avoid amino acid limitation that could potentially underestimate the biological response to ractopamine supplementation.

Under practical feeding conditions, diets used in combination with ractopamine are commonly formulated with increased amino acid density to match the elevated requirements associated with enhanced protein deposition (Almeida et al. 2010). Accordingly, the diets supplemented with ractopamine in this study were adjusted to provide nutritionally adequate conditions that allow the expression of the additive’s anabolic effects without imposing nutritional constraints.

The diets were pre-weighed for each pen and stored in labeled buckets placed in front of the experimental unit. To determine feed intake accurately, all feed refusals and wastage were collected daily and stored in plastic bags for weighing. Feed intake was recorded on days 4, 7, 11, 14, 17, and 21 to evaluate temporal variation during the experimental period. At the time of the second immunization (140 days of age), pigs had an average body weight of approximately 125.6 kg, which allowed the evaluation of growth performance during the final 21 days before slaughter.

The animals were individually weighed at the beginning and at the end of the experiment to determine final body weight, average daily gain, and feed conversion ratio, enabling a complete evaluation of growth performance during the 21-day period following the second immunization dose.

### In vivo carcass evaluation and *post-mortem* carcass evaluation

In vivo carcass assessment was performed using a Pig-Log^®^ 105 device (SFK Technology, Denmark) for measurement of backfat thickness (P1 and P2), loin depth, and estimated lean meat percentage. Hair was clipped at the measurement sites to standardize readings.

Point P1 was located 6.5 cm caudal to the last rib and 6.5 cm lateral to the dorsal midline (left side). Point P2 was positioned 6.5 cm cranial to the last rib and 6.5 cm lateral to the dorsal midline (left side). Loin depth was measured at P2. Lean meat percentage was automatically calculated by the equipment based on an internal equation incorporating loin depth and backfat thickness at P1 and P2. Measurements were obtained prior to slaughter to characterize treatment effects in vivo.

At the slaughter facility, carcass evaluation included hot carcass weight (HCW), carcass yield (CY), carcass length (CL), and backfat thickness at three anatomical sites, following Bridi and Silva (2009). HCW was recorded immediately after exsanguination and before chilling. CY was calculated as (HCW / final body weight) × 100.

Carcass length was measured from the cranial edge of the pubic symphysis to the cranio-ventral border of the atlas using a measuring tape. Backfat thickness was determined according to ABCS (1973) at three points, perpendicular to the dorsal midline: first rib (T1), tenth rib (T10), and last lumbar vertebra (TU). These measurements were used to evaluate fat deposition and treatment effects on final carcass traits.

### Statistical analysis

Data were analyzed using the MIXED procedure of SAS (version 9.4; SAS Institute Inc., Cary, NC, USA). The experiment followed a completely randomized 2 × 2 factorial design, including sex category (immunocastrated or surgically castrated males) and ractopamine level (0 or 10 ppm) as fixed effects. Means were separated using Tukey’s test.

For performance traits, the pen was considered the experimental unit. For in vivo and post-mortem carcass traits, one representative pig per pen was evaluated; therefore, each carcass observation corresponded to a unique experimental unit, avoiding non-independence among pigs housed in the same pen.

Because initial body weight differed among animals, it was included as a covariate in the performance models and removed when non-significant. Feed intake measured on days 4, 7, 11, 14, 17, and 21 was analyzed as repeated measures, with sex category, ractopamine level, period, and their interactions included as fixed effects. Non-significant interactions involving ractopamine × period and sex category × ractopamine × period were excluded from the final model. Statistical significance was declared at *P* < 0.05, and 0.05 ≤ *P* < 0.10 was considered a tendency.

## Results

**Fig. 1:**
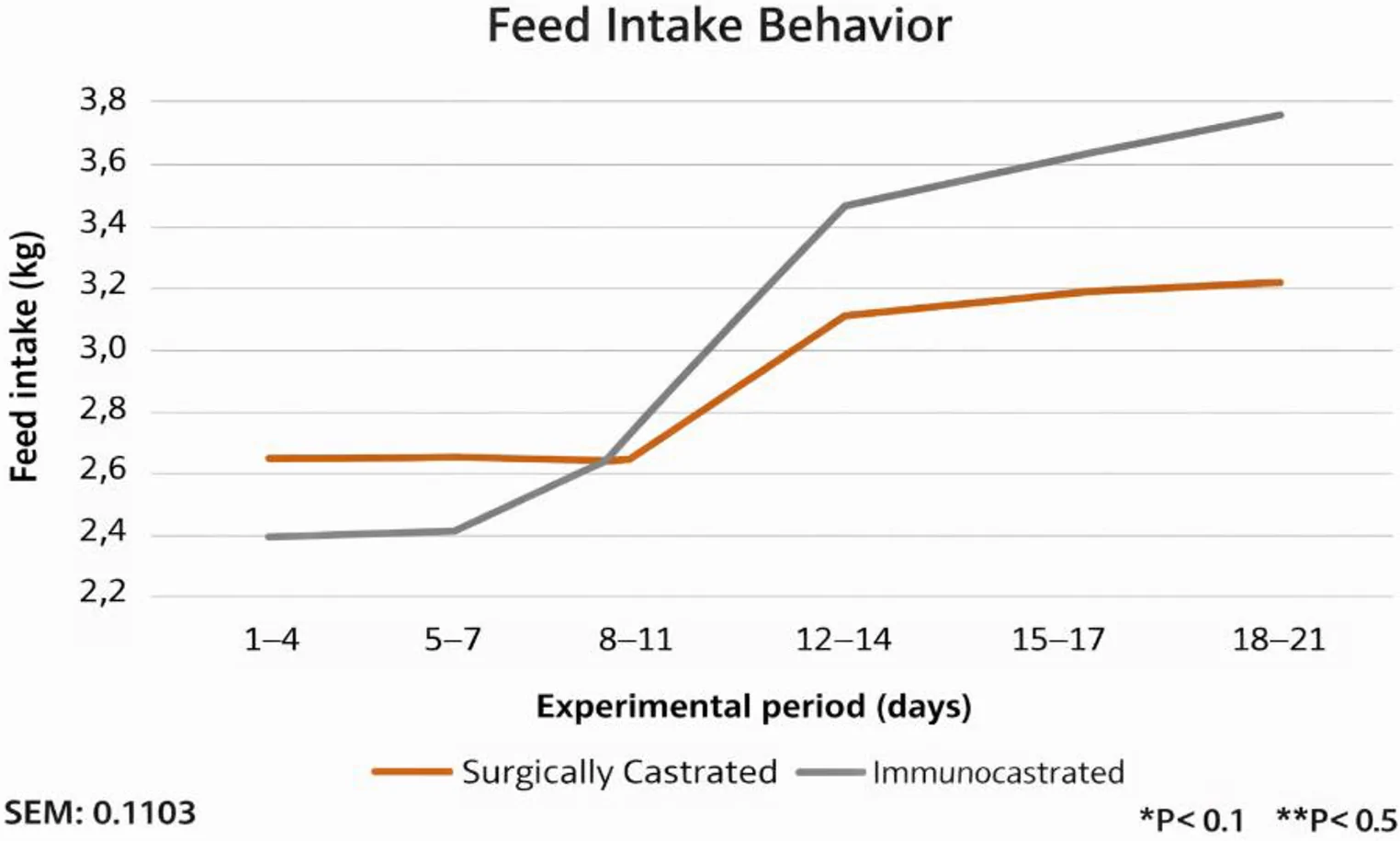
Feeding behavior of immunocastrated and surgically castrated male pigs uring the experimental periods (Periods 1–6) following the second immunization. Periods correspond to days 1–4 (Period 1), 5–7 (Period 2), 8–11 (Period 3), 12–14 (Period 4), 15–17 (Period 5), and 18–21 (Period 6).* Trend toward higher feed intake for immunocastrated pigs ** Higher feed intake for immunocastrated pigs

Immunocastration increased growth rate and improved feed efficiency in finishing males (Table 3). Immunocastrated pigs had higher ADG (19.5%; +191 g; *P* < 0.05) and greater final BW (*P* < 0.05), with a 13% reduction in FCR (*P* < 0.05). ADFI was not affected (*P* > 0.05). A sex category × period interaction was detected for ADFI, as immunocastrated pigs consumed more feed in Period 5 (+ 15.3%) and Period 6 (+ 18.5%; *P* < 0.05) (Fig. 1).

**Table 3:** Effect of sexual category and ractopamine supplementation on performance traits of pigs slaughtered at 145 days of age

Variables	Category (CT)		Ractopamine (RAC)		SEM	*P* value		
	SC	IM	0	10		CT	RAC	CT*RAC
Final body weight, kg	122.97 B	128.32 A	124.17 b	127.11 a	0.6038	< 0.0001	0.0018	0.8419
ADFI, kg/d	2.891	3.024	2.974	2.940	0.0861	0.2824	0.7825	0.8617
ADG, kg/d	0.980 B	1.171 A	1.007 b	1.144 a	0.0290	< 0.0001	0.0023	0.7892
FCR, kg/kg	2.977 B	2.594 A	2.982 b	2.590 a	0.0638	0.0002	0.0002	0.4506

¹ Uppercase letters in the same row differ (*P* < 0.05) for sexual category, and lowercase letters in the same row differ (*P* < 0.05) for ractopamine supplementation. 2 SEM: standard error of the mean. 3 SC: effect of sexual category. 4 RAC: ractopamine RS: effect of ractopamine supplementation

Ractopamine supplementation (10 ppm) also enhanced performance, increasing ADG (13.6%; +137 g; *P* < 0.05), reducing FCR by 13% (*P* < 0.05), and increasing final BW, without changing ADFI. No significant sex × ractopamine interaction was observed for any performance trait (*P* > 0.05).

For in vivo carcass traits (Table 4), immunocastrated pigs had lower backfat at P1 (− 33.8%) and P2 (− 28.8%) and greater loin depth (+ 11.1%; *P* < 0.05). After hair removal, ET1U (− 30.2%) and ET2U (− 27.8%) were reduced, and PCMU increased (+ 13.2%). Ractopamine did not affect in vivo carcass variables (*P* > 0.05). No sex category × ractopamine interaction was detected.

**Table 4:** Effect of sexual category and ractopamine supplementation on in vivo carcass traits of pigs slaughtered at 145 days of age

Variables	Category (CT)		Ractopamine (RAC)		SEM	*P* value		
	SC	IM	0	10		CT	RAC	CT*RAC
BF1D, mm	20.22 B	13.38 A	16.75	16.84	0.8800	< 0.0001	0.9405	0.3608
BF2D, mm	16.78 B	11.94 A	14.22	14.50	0.7184	< 0.0001	0.7839	0.2909
LMPD, %	52.67 B	58.53 A	55.75	55.46	0.7509	< 0.0001	0.7886	0.1246
BF1F, mm	23.48 B	16.38 A	20.66	19.20	0.8580	< 0.0001	0.2408	0.3812
BF2F, mm	21.28 B	15.38 A	18.75	17.90	0.7769	< 0.0001	0.4469	0.1041
LMPF, %	48.73 B	55.19 A	51.51	52.42	0.8077	< 0.0001	0.4323	0.1546

A-b: Uppercase letters in the same row differ (*P* < 0.05) for sexual category, and lowercase letters differ (*P* < 0.05) for ractopamine supplementation. ¹ BF1D: backfat thickness at point 1 measured on day 1 of the study; BF2D: backfat thickness at point 2 measured on day 1; ¹ LMPD: lean meat percentage measured on day 1; BF1F: backfat thickness at point 1 measured on the final day; BF2F: backfat thickness at point 2 measured on the final day; LMPF: lean meat percentage measured on the final day. 2 SEM: standard error of the mean. 3 CT: effect of sexual category. 4 RAC: effect of ractopamine supplementation

Post-mortem, carcass yield was higher in surgically castrated pigs (+ 3.4%; *P* < 0.05) (Table 5). Immunocastrated pigs had lower backfat at T1 (− 10.5%), T10 (− 23.5%), and TU (− 21.5%). Ractopamine increased HCW by 3.1% (*P* < 0.05) and did not affect CY, CL, or backfat traits (*P* > 0.05). No interaction was observed between sex category and ractopamine.

**Table 5:** Effect of sexual category and ractopamine supplementation on *post-mortem* carcass characteristics of pigs slaughtered at 145 days of age

Variables	Category (CT)		Ractopamine (RAC)		SEM	*P* value		
	SC	IM	0	10		CT	RAC	CT*RAC
HCW, kg	99.89	100.68	98.75 b	101.81 a	0.4989	0.2740	0.0002	0.9027
CY, %	81.24 A	78.56 B	79.67	80.13	0.0019	< 0.0001	0.0999	0.7173
CC, cm	104.19	104.39	104.73	103.86	0.6604	0.8304	0.3616	0.3364
BF1, cm	4.11 B	3.68 A	3.88	3.92	0.1008	0.0053	0.7741	0.3770
BF10, cm	2.83 B	2.16 A	2.53	2.46	0.0874	< 0.0001	0.6087	0.6541
BFU, cm	2.56 B	2.01 A	2.31	2.27	0.0920	0.0003	0.7603	0.1534

A-b: Uppercase letters in the same row differ (*P* < 0.05) for sexual category, and lowercase letters differ (*P* < 0,05) for ractopamine supplementation. ¹ HCW: hot carcass weight; CY: carcass yield; CC: carcass length; BF1: backfat thickness at point 1; BF10: backfat thickness at point 2; BFU: backfat thickness at point 3. 2SEM: standard error of the mean. 3CT: effect of sexual category. 4RAC: effect of ractopamine supplementation

## Discussion

Although ractopamine rapidly enhances protein synthesis and feed efficiency, changes in carcass traits generally require longer exposure periods. Therefore, a 21-day supplementation period may be sufficient to elicit metabolic and performance responses, but not long enough for pronounced structural changes in carcass composition, as reported in studies using longer feeding durations.

The performance outcomes observed in the present study reinforce the superior productive efficiency of immunocastrated (IM) pigs compared with surgically castrated (SC) pigs and demonstrate that the 21-day window following the second vaccine dose remains metabolically active and conducive to accelerated growth. Immunocastrated pigs exhibited a 19.5% higher ADG, a response comparable to the 23% improvement described by Puls et al. (2014). Even with the shorter interval between the second vaccination and slaughter adopted in the present study, growth acceleration was maintained. This response is likely related to the prolonged exposure to endogenous androgens prior to the second dose, which sustains higher rates of protein deposition and muscle accretion (Metz and Claus 2003; Čandek-Potokar et al. 2017). This growth advantage is consistent with previous reports describing superior productive performance in immunocastrated pigs during the period immediately following the second vaccination (Poulsen Nautrup et al. 2018).

Surgically castrated pigs exhibited lower feed efficiency compared with the other categories. Silva-Santos et al. (2024) reported FCR values 0.27 kg/kg higher than those of intact males and 0.22 kg/kg higher than entire males. In the present study, although SC pigs consumed slightly more feed (approximately 0.23 kg/day above IM pigs), this additional intake did not translate into greater weight gain. In fact, ADG was reduced by 9.54 g/day relative to immunocastrated animals, reflecting a lower capacity to convert nutrients into lean tissue.

The 13% improvement in FCR observed in immunocastrated pigs agrees with previous findings (Fàbrega et al. 2010; Demori et al. 2015). Rather than differences in total feed intake, the enhanced efficiency appears to be associated with a shift in nutrient partitioning, favoring lean deposition over fat accumulation.

By maintaining the physiological profile of entire males for most of the production cycle, immunocastrated pigs benefit from continuous secretion of anabolic hormones such as testosterone until administration of the second vaccine dose (Han et al. 2019).The persistence of circulating androgens prior to the second immunization contributes to enhanced protein accretion and accelerated muscle growth, which ultimately translates into improved feed efficiency. Following the booster dose, endocrine profiles resemble those of surgically castrated pigs (Čandek-Potokar et al. 2017). Nevertheless, immunocastrated animals continue to display a productive advantage, as reported by Poulsen Nautrup et al. (2018) and Dalla Costa et al. (2020).

Across evaluation periods, immunocastrated pigs showed a marked increase in feed intake during days 18–21 and a tendency toward higher consumption in the preceding phase, suggesting the beginning of post-vaccination hyperphagia. Elsbernd et al. (2017) described a comparable pattern, with increased intake occurring approximately 13–14 days after the second dose. In the present trial, however, overall average daily intake remained unchanged. This may indicate that the 21-day interval between the second immunization and slaughter was not long enough for full expression of hyperphagia. Under these conditions, productive responses were evident within 21 days after the second immunization. However, direct comparisons with longer post-immunization intervals were beyond the scope of the present study and require further investigation. Within this context, the effects of ractopamine supplementation on growth performance and carcass traits deserve further discussion.

The productive responses observed with RAC supplementation should be interpreted within the nutritional strategy adopted in this study. Diets containing RAC were formulated with higher crude protein and digestible amino acid concentrations to meet the increased requirements associated with enhanced protein deposition. Previous studies have shown that the effectiveness of ractopamine depends on an adequate amino acid supply, particularly lysine, and responses may be attenuated when these nutritional requirements are not met (Apple et al. 2004; Almeida et al. 2010). Therefore, the improvements observed in ADG and feed efficiency likely reflect the combined effect of β-adrenergic stimulation and the nutritional support required to maximize protein accretion, which is consistent with the productive responses reported in studies evaluating ractopamine under nutritionally adequate feeding programs.

In practical terms, the 13.6% gain in ADG and the parallel improvement in feed conversion are consistent with β-adrenergic agonists (Moody et al. 2000). Because feed intake did not differ, the response appears to be associated with improved nutrient utilization and muscle protein accretion rather than increased consumption (Gerlemann et al. 2014; Abbas et al. 2022). The productive responses observed after 21 days of ractopamine supplementation are comparable to those described by Rosa (2011), Garbossa et al. (2013), and Leal et al. (2015), indicating that this duration is sufficient to promote measurable improvements in performance. Kutzler et al. (2010) reported a more pronounced effect on feed conversion (approximately 20%); however, Feldpausch et al. (2016) documented responses closer to those obtained in the present study. Together, these results suggest that the magnitude of RAC response is influenced by factors such as genotype, environmental conditions, and dietary formulation.

Evidence from previous trials helps contextualize the performance advantage observed in immunocastrated pigs. Brustolini et al. (2019) reported that immunocastrated animals receiving ractopamine reached an ADG of 1.296 kg/day, whereas restricted surgically castrated pigs without RAC achieved 0.945 kg/day. Likewise, Pérez-Ciria et al. (2022) described improved feed efficiency in immunocastrated males. In the present study, the lack of interaction between ractopamine and sex category suggests that each strategy operates through distinct physiological pathways, resulting in additive rather than synergistic responses. From a practical perspective, this allows both approaches to be applied concurrently in heavy-weight production systems without detrimental effects on growth performance (Lowe et al. 2014).

The absence of significant differences in total feed intake, despite the effects observed during periods 5 and 6, is likely related to the transient physiological changes that occur after the second immunization. Following the second vaccination, immunocastrated pigs undergo a rapid suppression of testicular function, resulting in a marked reduction in circulating testosterone. This endocrine shift has been consistently associated with increased appetite and voluntary feed intake, which explains the higher feed consumption observed during this specific post-immunization window (Elsbernd et al. 2017; Morales et al. 2017).

However, because this response is concentrated over a relatively short period, its impact may be diluted when feed intake is evaluated cumulatively across the entire experimental period, leading to non-significant differences in total feed intake. Similar time-dependent patterns of feed intake after the second immunization have been reported in the literature, indicating that changes in feeding behavior are not sustained throughout the finishing phase (Schiavon et al. 2022).

The reduction in subcutaneous fat observed in immunocastrated pigs in line with previous reports. Demori et al. (2015), for example, describe a 17.1% decrease in backfat thickness at the 10th rib in this category. From a commercial standpoint, this lower fat deposition is particularly relevant for markets that prioritize leaner carcasses and higher processing efficiency. In the present study, the greater lean meat percentage likely reflects the prolonged anabolic stimulus before the second vaccine dose and a more efficient use of amino acids for muscle protein deposition. In contrast, surgically castrated pigs appear more prone to channel surplus energy toward lipid accretion, as previously discussed by Van den Broeke et al. (2017). Our results align with those of Moraes et al. (2009), who also documented greater lean meat deposition in immunocastrated pigs, indicating that this response has been consistently observed under different production conditions.

Conversely, ractopamine did not affect most carcass traits within the 21-day supplementation period evaluated in the present study. Similar findings were reported by Rosa (2011), whereas Marinho et al. (2007) observed more pronounced =carcass responses with longer supplementation periods. In that study, ractopamine increased loin depth by 4.9% after 21 days and by 6.5% after 28 days, while backfat thickness at P2 was reduced by approximately 8.1% after 28 days of supplementation. These comparisons indicate that more pronounced carcass responses have frequently been reported in studies evaluating longer supplementation periods, whereas the present study detected minimal carcass responses despite clear improvements in growth performance.

Hot carcass weight did not differ between immunocastrated and surgically castrated pigs, which agrees with Caldara et al. (2013), Asmus et al. (2014), Boler et al. (2013), Morales et al. (2014), and Lowe et al. (2014). Although immunocastration alters tissue composition, the shorter interval between the second dose and slaughter appears insufficient to significantly change absolute carcass weight (Božičković et al. 2026).

The higher carcass yield observed in SC pigs is consistent with previous reports comparing immunocastrated and surgically castrated males (Morales et al. 2014). Although organ and reproductive tract weights were not measured in the present study, previous studies have reported greater visceral organ development and heavier reproductive tissues in immunocastrated pigs due to their prolonged exposure to anabolic hormones before the second immunization (Batorek et al. 2012; Čandek-Potokar et al. 2017). These factors may contribute to the lower dressing percentage commonly observed in immunocastrated animals.

In addition, a tendency toward higher carcass yield was observed with ractopamine supplementation (*P* = 0.0999), which is consistent with previous reports showing modest improvements in carcass efficiency associated with β-adrenergic agonists, even when statistical significance is not achieved.

Ractopamine supplementation increased hot carcass weight by 3.1%, similar to the 4.1% reported by Rickard et al. (2017) using 7.4 ppm. This result is supported by Gerlemann et al. (2014) and is explained by the metabolic repartitioning induced by RAC (Feldpausch et al. 2016). Although ractopamine supplementation increased hot carcass weight, carcass yield itself remained unchanged. Similar outcomes were described by Oliveira et al. (2013) and Leal et al. (2015). This response is expected, as RAC promotes greater skeletal muscle accretion while having little to no influence on the relative weight of visceral organs (Mersmann 2002). Finally, the carcass values observed in immunocastrated pigs, HCW of 88.66 kg, carcass yield up to 78.5%, and high yields of loin (0.700 kg), shoulder (19.76%), and ribs (7.09%), reinforce their potential as a category that balances production efficiency and commercial value of cuts (Lima et al. 2025).

## Conclusion

Immunocastration improved growth performance and carcass leanness in finishing male pigs, even within the shortened 21-day interval. Immunocastrated pigs showed higher daily gain, better feed efficiency, and reduced fat deposition, confirming the metabolic responsiveness of this period. Pigs fed diets supplemented with ractopamine (10 ppm) under nutritionally adjusted feeding conditions designed to support increased protein deposition showed greater average daily gain and increased hot carcass weight, without interaction between technologies. Both strategies acted independently and additively, indicating that ractopamine supplementation within a nutritionally adequate feeding strategy can improve growth performance and carcass efficiency in finishing pigs.

## Data Availability

The data that support the findings of this study are available on request from the corresponding author.

## References

[CR1] Abbas K, Raza A, Vasquez RD, Roldan MJMM, Malhotra N, Huang JC, Buenafe OEM, Chen KHC, Liang SS, Hsiao CD (2022) Ractopamine at the center of decades-long scientific and legal disputes: A lesson on benefits, safety issues, and conflicts. Biomolecules 12:1342 10.3390/biom12101342 PMC9599871 36291550

[CR8] ABCS - Associação Brasileira de Criadores de Suínos (1973) Métodos brasileiros de classificação de carcaças, 2nd edn. Estrela

[CR2] Abreu RC, Oliveira BF, Rodrigues GP et al (2024) Chromium and feed restriction as alternative strategies to modulate finishing pig performance and carcass. Ciência Rural 54:e20220673

[CR3] Adeola O, Darko EA, He P, Young LG (1990) Manipulation of porcine carcass composition by ractopamine. J Anim Sci 68:3633–3641. 10.2527/1990.68113633x 1979784

[CR5] Almeida EC, Fialho ET, Rodrigues PB, Zangeronimo MG, Lima JAF, Fontes DO (2010) Ractopamine and lysine levels on performance and carcass characteristics of finishing pigs. Revista Brasileira de Zootecnia 39:1961–1968

[CR6] Apple JK, Maxwell CV, Brown DC, Friesen KG, Musser RE, Johnson ZB, Armstrong TA (2004) Effects of dietary lysine and energy density on performance and carcass characteristics of finishing pigs fed ractopamine. J Anim Sci 82:3277–3287. 10.2527/2004.82113277x 15542474

[CR7] Asmus MD, Tavarez MA, Tokach MD, Dritz SS, Schroeder AL, Nelssen JL, Goodband RD, DeRouchey JM (2014) The effects of immunological castration and corn dried distillers grains with solubles withdrawal on growth performance, carcass characteristics, fatty acid analysis, and iodine value of pork fat depots. J Anim Sci 92:2116–2132 10.2527/jas.2013-6910 24668958

[CR9] Batorek N, Čandek-Potokar M, Bonneau M, Van Milgen J (2012) Meta-analysis of the effect of immunocastration on production performance, reproductive organs and boar taint compounds in pigs. Animal 6:1330–1338 10.1017/S1751731112000146 23217237

[CR10] Boler DD, Puls CL, Clark DL, Ellis M, Schroeder AL, Matzat PD, Killefer J, McKeith FK, Dilger AC (2013) Effects of immunological castration (Improvest) on changes in dressing percentage and carcass characteristics of finishing pigs. J Anim Sci 91:359–368 10.2527/jas.2013-6863 24243892

[CR55] Božičković I, Radojković D, Savić R, Popovac M, Brun A, Soler J, Lizardo R, Font-i-Furnols M (2026) Bones, reproductive organs and carcass characteristics of entire, early and late immunocastrated male pigs. Animal 20:101726. 10.1016/j.animal.2025.101726 41389400

[CR12] Bridi AM, Silva CA (2009) Avaliação da carne suína, 1st edn. Midiograf, Londrina

[CR13] Brustolini APL, Rodrigues LA, Silva FCO, Peloso JV, Aldaz A, Junior MBC, Figueiredo TC, Alkimin DV, Fontes DO (2019) Interactive effects of feed allowance and ractopamine supplementation on growth performance and carcass traits of physically and immunologically castrated heavy weight pigs. Livest Sci 228:120–126. 10.1016/j.livsci.2019.08.009

[CR15] Čandek-Potokar M, Škrlep M, Zamaratskaia G (2017) Immunocastration as alternative to surgical castration in pigs. Theriogenology 6:109–126. 10.5772/intechopen.68650

[CR14] Caldara FR, Moi M, Santos LSS, Paz ICLA, Garcia RG, Nääs IA, Fernandes ARM (2013) Carcass characteristics and qualitative attributes of pork from immunocastrated animals. Asian-Australasian J Anim Sci 26:1630–1636 10.5713/ajas.2013.13160 PMC4093819 25049751

[CR17] Dalla Costa OA, Tavernari FC, Lopes LS, Dalla Costa FA, Feddern V, Lima GJM (2020) Performance, carcass and meat quality of pigs submitted to immunocastration and different feeding programs. Res Vet Sci 131:137–145. 10.1016/j.rvsc.2020.04.015 32360912

[CR18] Demori AB, Andretta I, Kipper M, Lanferdini E, Lehnen CR (2015) Produção de suínos machos em crescimento: Uma meta-análise. Revista Brasileira de Saúde e Produção Anim 16:130–138

[CR19] Elsbernd AJ, Lange CFM, Stalder KJ, Karriker LA, Patience JF (2017) SID lysine requirement of immunologically and physically castrated male pigs during the grower, early and late finisher periods. J Anim Sci 95:1253–1263 10.2527/jas.2016.0544 28380505

[CR20] Fàbrega E, Velarde A, Cros J, Gispert M, Suárez P, Tibau J, Soler J (2010) Effect of vaccination against gonadotrophin-releasing hormone, using Improvac^®^, on growth performance, body composition, behaviour and acute phase proteins. Livest Sci 132:53–59

[CR21] Feldpausch JA, Amachawadi RG, Tokach MD, Scott HM, Nagaraja TG, Dritz SS, Goodband RD, Woodworth JC, DeRouchey JM (2016) Effects of dietary copper, zinc, and ractopamine hydrochloride on finishing pig growth performance, carcass characteristics, and antimicrobial susceptibility of enteric bacteria. J Anim Sci 94:3278–3293 10.2527/jas.2016-0340 27695810

[CR23] Garbossa CAP, Sousa RV, Cantarelli VS, Pimenta MESG, Zangeronimo MG, Silveira H, Kuribayashi TH, Cerqueira LGS (2013) Ractopamine levels on performance, carcass characteristics and quality of pig meat. Revista Brasileira de Zootecnia 42:325–333

[CR24] Gerlemann GD, Allee GL, Rincker PJ, Ritter MJ, Boler DD, Carr SN (2014) Impact of ractopamine hydrochloride on growth, efficiency, and carcass traits of finishing pigs in a three-phase marketing strategy. J Anim Sci 92:1200–1207 10.2527/jas.2013-6548 24492553

[CR25] Han X, Zhou M, Cao X, Du X, Meng F, Bu G (2019) Mechanistic insight into the role of immunocastration on eliminating skatole in boars. Theriogenology 131:32–40. 10.1016/j.theriogenology.2019.03.017 30939354

[CR26] Kutzler LW, Peterson CM, Ellis M, Carr SN, Ritter MJ, Armstrong TA, McKeith FK, Killefer J (2010) Ractopamine (Paylean) response in heavy-weight finishing pigs. Prof Anim Sci 26:243–249

[CR27] Leal RS, Mattos BO, Cantarelli VS, Carvalho GC, Pimenta MESG, Pimenta CJ (2015) Desempenho e rendimento de carcaça de suínos na fase de terminação recebendo dietas com diferentes níveis de ractopamina. Revista Brasileira de Saúde e Produção Anim 16:582–589

[CR28] Lealiifano AK, Pluske JR, Nicholls RR, Dunshea FR, Campbell RG, Hennessy DP, Miller DW, Hansen CF, Mullan BP (2011) Reducing the length of time between slaughter and the secondary gonadotropin-releasing factor immunization improves growth performance and clears boar taint compounds in male finishing pigs. J Anim Sci 89:2782–2792. 10.2527/jas.2010-3267 21512121

[CR56] Lima MD, Ferreira SV, Lopes IMG, Ramos LA, Silveira NCS, Santos LDT, Figueiredo TC, Fontes DO (2025) Feeding programs for immunocastrated male and female Duroc hybrid pigs in growing and finishing phases. Meat Sci 228:109872. 10.1016/j.meatsci.2025.109872 40516218

[CR29] Lowe BK, Gerlemann GD, Carr SN, Rincker PJ, Schroeder AL, Oetry DB, McKeith FK, Allee GL, Dilger AC (2014) Effects of feeding ractopamine hydrochloride to physical and immunological castrates in a commercial setting on growth performance and carcass characteristics. J Anim Sci 92:3727–3735 10.2527/jas.2013-7516 25006070

[CR30] Marinho PC, Fontes DO, Silva FCO, Silva MA, Pereira FA, Arouca CLC (2007) Efeito da ractopamina e de métodos de formulação de dietas sobre o desempenho e as características de carcaça de suínos machos castrados em terminação. Revista Brasileira de Zootecnia 36:1061–1068

[CR31] Mersmann HJ (2002) Beta-adrenergic receptor modulation of adipocyte metabolism and growth. J Anim Sci 80:E24–E29. 10.2527/animalsci2002.0021881200800ES10005x

[CR32] Metz C, Claus R (2003) Active immunization of boars against GnRH does not affect growth hormone but lowers IGF-I in plasma. Livest Prod Sci 81:129–137

[CR33] Moody DE, Hancock DL, Anderson DB (2000) Phenethanolamine repartitioning agents. In: D’Mello JPF (ed) Farm animal metabolism and nutrition. CABI, pp 65–95

[CR34] Moraes E, Ciefer C, Silva IS (2009) Ractopamina em dietas para suínos machos imunocastrados, castrados e fêmeas. Ciência Rural 39:231–237

[CR36] Morales JI, Serrano MP, Cámara L, Berrocoso JD, López JP, Mateos GG (2014) Growth performance and carcass quality of immunocastrated and surgically castrated pigs. J Anim Sci 91:3955–3964 10.2527/jas.2012-6068 23658332

[CR35] Morales J, Dereu A, Manso A, Frutos L, Piñeiro C, Manzanilla EG, Wuyts N (2017) Surgical castration with pain relief affects the health and productive performance of pigs in the suckling period. Porcine Health Manage 3:1–10. 10.1186/s40813-017-0066-1 PMC5585944 28879020

[CR37] Mustafa RJ, Khaleel AW, Ezzulddin TA (2026) Molecular Technologies in Animal Improvement. Istanus J Appl Biol Sci 2(1):e3. 10.64012/istanusjabs.2026.2.1.e3

[CR38] Oliveira BF, Kiefer C, Santos TMB, Garcia ERM, Marçal DA, Abreu RC, Rodrigues GP (2013) Período de suplementação de ractopamina em dietas para suínos machos castrados em terminação. Ciência Rural 43:355–360

[CR54] Pérez-Ciria L, Miana-Mena FJ, Álvarez-Rodríguez J, Latorre MA (2022) Effect of castration type and diet on growth performance, serum sex hormones and metabolites, and carcass quality of heavy male pigs. Animals 12:1004. 10.3390/ani12081004 PMC9024496 35454250

[CR52] Poulsen Nautrup B, Van Vlaenderen I, Aldaz A, Mah CK (2018) The effect of immunization against gonadotropin-releasing factor on growth performance, carcass characteristics and boar taint relevant to pig producers and the pork packing industry: a meta-analysis. Res Vet Sci 119:182–195. 10.1016/j.rvsc.2018.06.002 29958153

[CR39] Puls CL, Rojo A, Ellis M, Boler DD, McKeith FK, Killefer J, Gaines AM, Matzat PD, Schroeder AL (2014) Growth performance of immunologically castrated barrows compared to gilt, physically castrated barrow, and intact male pigs. J Anim Sci 92:2289–2295 10.2527/jas.2013-6861 24671576

[CR40] Rickard JW, Allee GL, Rincker PJ, Gooding JP, Acheson RJ, McKenna DR, Puls CL, Carr SN (2017) Effects of ractopamine hydrochloride on growth performance and carcass characteristics of heavy-weight finishing pigs. Translational Anim Sci 1:406–411 10.2527/tas2017.0053 PMC7205354 32704664

[CR41] Rosa BO (2011) Níveis de lisina digestível e de ractopamina para suínos machos imunocastrados em terminação. Dissertação, Universidade Federal de Minas Gerais, Belo Horizonte

[CR42] Rossi GP et al (2022) Does immunocastration affect behaviour and body composition parameters in male pigs compared to surgical castration? J Vet Pharmacol Ther

[CR43] Rostagno HS, Albino LFT, Hannas MI, Sakomura NK, Perazzo FG, Saraiva A, Abreu MLT, Rodrigues PB, Barreto SLT, Brito CO (2017) Tabelas brasileiras para aves e suínos: composição de alimentos e exigências nutricionais, 4th edn. Universidade Federal de Viçosa

[CR44] Sánchez-Villalba E, López-Vergara V, Pérez-Ramírez H, Morales-León L, García-Hernández R, Rocha-Martínez J, Flores-Peinado S, Sánchez-Hernández MA, Martínez-Rocha J, Zamora-Gasca VM (2023) Effects of chromium methionine and ractopamine supplementation on performance, carcass traits and meat quality of finishing pigs under heat stress. Animals 13:2671

[CR45] Sardi L, Martelli G, Lambertini L, Parisini P, Mordenti AL (2020a) Effects of pre-slaughter conditions on carcass traits and meat quality in heavy pigs. Animals 10:945. 10.3390/ani10060945

[CR46] Sardi L, Martelli G, Lambertini L, Parisini P, Mordenti AL (2020b) Pre-slaughter stress and its impact on meat quality traits in heavy pigs. Animals 10:2386. 10.3390/ani10122386

[CR48] Silva-Santos HR, Martínez-Castañeda FE, Álvarez-Fuentes G, Rubio-Lozano MS, Trujillo-Ortega ME (2024) Effect of various castration protocols on production indicators in pigs: Meta-analysis. Revista Mexicana de Ciencias Pecuarias 15:267–286. 10.22319/rmcp.v15i2.6420

[CR47] Schiavon S, Malgwi IH, Giannuzzi D, Galassi G, Rapetti L, Carnier P, Halas V, Gallo L (2022) Impact of rearing strategies on metabolizable energy and lysine partitioning in pigs. Animals 12:689. 10.3390/ani12060689 PMC8944463 35327086

[CR53] Van den Broeke A, Leen F, Aluwé M, Van Meensel J, Millet S (2017) Effect of slaughter weight and sex on carcass composition and N- and P-efficiency of pigs. In: Book of Abstracts of the 68th Annual Meeting of the European Association for Animal Production. Tallinn, p 111.

